# A long-term cohort study of surgery for recurrent prolapse comparing mesh augmented anterior repairs to anterior colporrhaphy

**DOI:** 10.1186/s10397-017-1035-z

**Published:** 2018-01-10

**Authors:** Natasha Curtiss, Jonathan Duckett

**Affiliations:** Department of Obstetrics and Gynaecology, Medway Maritime Hospital, Windmill Road, Gillingham, Kent ME7 5NY UK

**Keywords:** Mesh, Perigee, Pelvic organ prolapse, Surgery, Colporrhaphy, Long term

## Abstract

**Background:**

There are safety concerns regarding the use of mesh in vaginal surgery with a call for long-term follow-up data. This study was designed to evaluate the long-term safety and efficacy of vaginal repairs performed for recurrent cystocele using Perigee (non-absorbable trans-obturator) mesh.

**Methods:**

A retrospective consecutive cohort of 48 women who underwent surgery for recurrent prolapse between March 2007 and December 2011 in a single centre was reviewed. Satisfaction was assessed using the patient global impression of improvement (PGI-I). Symptoms were assessed with the pelvic floor distress inventory (PFDI). Women were questioned regarding pain, sexual activity and pelvic floor surgery performed since the original procedure and examined for erosion. Women were compared to 25 controls from a consecutive cohort of repeat anterior colporrhapies.

**Results:**

The mean length of follow-up was 6.5 years (78 months; range 48–106). Significantly more women in the mesh group reported that they were “much better” or “very much better” (69 vs 40% *p* = 0.02). The rate of mesh erosion at follow-up was 11.6%. Two women in the mesh group required surgical excision of eroded mesh in the operating room (4%). The reoperation rate for a combination of de novo stress incontinence, recurrent prolapse and mesh exposure was similar in each group (33% mesh vs 32% native tissue).

**Conclusions:**

A vaginal mesh repair using a non-absorbable trans-obturator mesh has improved satisfaction compared to an anterior colporrhaphy.

## Background

Pelvic organ prolapse (POP) is a common condition with considerable socio-economic, psychological and physical impact [1, 2]. Eleven percent of women will have undergone a surgical repair by the age of 80 [3]. The most common repair to be performed is an anterior vaginal wall repair [4].

There is a significant recurrent prolapse rate after primary native tissue repair [5]. Mesh repairs were introduced to reinforce the native tissues aiming to reduce recurrence rates. A Cochrane review suggested recurrence rates are lower using non absorbable mesh augmented repairs [6]. However, there are complications unique to mesh repairs and 12% of patient will have a mesh complication and a proportion of these will require further surgical intervention to manage the complication [5, 6]. High rates of pain and dyspareunia have also been reported affecting patient satisfaction [7].

The reported complications of mesh-augmented repairs have resulted in interest from regulatory bodies including the FDA, the media and the patient support groups. The Scottish Government ordered an independent review of the use of transvaginal mesh in women and NHS England produced an interim report in October 2015 [8]. The long-term safety and prolapse recurrence is of concern to urogynecologists and their women, and there is currently a paucity of long-term evidence. This study aimed to look at the long-term safety and efficacy of vaginal repairs performed using Perigee mesh for recurrent cystocele and compare them to native tissue repair/anterior colporrhaphy for recurrent cystocele. This was a single-center single-surgeon cohort study.

## Methods

All women who had undergone a recurrent cystocele (symptomatic POP-Q stage 2) repair using either native tissue or a Perigee vaginal mesh repair at a single surgical centre were identified from a prospectively maintained database. This was cross-referenced with information from the British Society of Urogynaecology (BSUG) database. All procedures were performed by or under the supervision of a single surgeon. All procedures were carried out in an operating theatre under regional or general anaesthesia with administration of prophylactic antibiotics. Fascial plication was performed in the native tissue repairs using absorbable polyglactin sutures. A continuous locking polyglactin suture was used for vaginal skin closure. The same technique was used for mesh repairs other than the repair was augmented with placement of non-absorbable mesh under the fascial plication sutures. The mesh was placed in a tension-free fashion with the aim of supporting the whole of the anterior vaginal wall. The upper transobturator arm was placed 1/2 cm in front of the ischial spine. Women with vault prolapse (greater than or equal to stage 2) were treated with abdominal sacrocolpopexy and were excluded from this study.

A consecutive cohort of women receiving Perigee mesh vaginal repairs for recurrent cystoceles between March 2007 and December 2011 was contacted and asked to attend for clinical assessment. The time frame and number of women were purely dependent on the number of operations performed. A post hoc power calculation suggested that a study with 79 recruits had a 70% chance of demonstrating a difference in the patient global impression of improvement (PGI-I) with a *p* value of 0.05. Before 2007, mesh kits were not used. After 2011, very few meshes were inserted into the anterior vaginal wall due to concerns regarding safety and efficacy. No other anterior vaginal wall mesh kit was used before, during or after these dates. Meshes were not inserted for primary repairs. Demographic data and details regarding the surgery were gathered from the patient’s clinical record. The primary outcome was based on the PGI-I (much better and very much better) with the presence of prolapse symptoms ascertained by the pelvic floor distress inventory (PFDI). Women were interviewed regarding their awareness of mesh complications reported in the media. Women were asked if they were sexually active before the surgery and whether they continued to be sexually active at follow-up. Women were examined for mesh exposure. Women were assessed as having a mesh erosion if one was detected on examination or if the patient had undergone medical or surgical treatment for mesh erosion during the follow-up period. A single research fellow who was not previously known by the women undertook the clinical examinations. This person assessed the clinical notes and hence was not blinded to the previous surgery. Women who did not attend for clinical assessment were contacted by telephone, where possible, questionnaires and interviews were undertaken over the telephone.

Women who had undergone native tissue repairs at the same centre for recurrent cystocele during the same timespan completed the same questionnaires and were contacted and interviewed by telephone. Their notes were scrutinised, for demographic and operative details. There were limited cases in this cohort as women were primarily offered mesh repairs during this time. The same time frame was used so that women with a similar length of follow-up would be studied.

A symptomatic recurrence was assessed when either further surgery had been undertaken or if the woman had answered Somewhat, Moderately or Quite a Bit to the PFDI-6 question “usually have a bulge or something falling out that you can see or feel in your vaginal area”. All definitions and terminology confirm to the joint International Urogynecology Association/International Continence Society terminology report [9]. *T* tests were used for continuous variables which were normally distributed, and proportions were compared using the Chi-squared test.

### Ethical consent and permissions

Ethical approval for the study was given by the National Research Ethics Service (NRES) (Ref 15/LO/0158: 6th February 2015), and the study was registered with ClinicalTrials.gov (NCT02642835). Patients provided written informed consent. The research was performed in accordance with the Helsinki declaration.

## Results

**Fig. 1:**
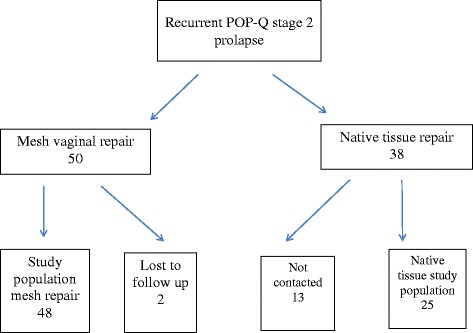
Patient flow

A total of 50 women were identified as having had a Perigee mesh vaginal repair for recurrent cystocele in the study period (see Fig. 1). Forty-eight women were assessed (five by telephone interview). Two women were lost to follow-up. One had died from unrelated causes. There were 38 women identified as having undergone a native tissue repair for recurrent cystocele in the same time period. Twenty-five were interviewed (66%) and one woman had died of unrelated causes. The mean length of follow-up was 6.5 years (78 months; range 48–106). There was no difference in the patient demographics (see Table 1). The population was predominantly white Caucasian (98%). There were no intra-operative complications in either group. No woman required a blood transfusion. The outcome data is presented in Table 2. Nine women (19%) interviewed in the Perigee group reported being aware of concerns regarding vaginal mesh in the media or from friends or family prior to receiving the invitation to be part of the study.

**Table 1:** Demographic data

	Perigee (*n* = 48)	Native tissue (*N* = 25)	*P* value
Age (range)	66 (47–83)	62 (44–81)	0.10
BMI (mean)	27.4	30.0	0.12
Parity (median)	2	3	
Smoking (current)	7	4	1
Time to follow-up (months) (range)	82 (48–106)	69 (53–101)	0.01
Prior hysterectomy	98% (47/48)	88% (22/25)	0.11

**Table 2:** Post operative outcomes

	Perigee (*n* = 48)	Native tissue (*N* = 25)	*P* value
Much or very much better PGI-I	33 (68.8)	10 (40%)	0.02
Sexually active preoperatively	37 (75.5%)	15 (60%)	0.0002
Stopped sexually active after most recent repair	17/37 (46%)	6/15 (40%)	0.77
Operation for SUI during follow-up	5 (10.4%)	2 (8%)	1
Reoperation for POP: same compartment	1 (2.1%)	2 (8%)	0.29
Reoperation different compartment	8 (16.7%)	4 (16%)	
Symptomatic recurrence (PFDI)	14 (29.2%)	13 (52%)	0.08
Surgery for mesh erosion	2 (4%)	0	N/A

*SUI* stress urinary incontinence, *PGI-I* patient impression of improvement, *POP* pelvic organ prolapse, *PFDI* pelvic floor distress inventory

Significantly more women in the mesh group reported that they were “much better” or “very much better” (69 vs 40% *p* = 0.02). In the Perigee group, the rate of mesh erosion at follow-up was 11.6% (5/43). None of these occurred in either women with diabetes 3/48 or in smokers 7/48. Two erosions were treated with topical oestrogen alone, one was cut in clinic (IUGA graft complication code 2AT4S1) and the other two required surgical excision in the operating theatre (2/48; 4%) (3AT4S2). Three of the mesh erosions were identified for the first time during the study; two had symptoms of prolapse recurrence and the other overactive bladder symptoms but none had re-presented before invitation with these symptoms. These women were unaware of the mesh exposure and did not have exposure-specific symptoms. Mesh erosion is a complication that does not affect native tissue repairs. Table 3 describes the surgical procedures that the women had undergone since the index procedure. When the need for repeat surgery for mesh exposure, surgery for de novo stress incontinence and repeat surgical treatment of prolapse are combined 33% (16/48) of the Perigee group and 32% (8/25) of the native tissue repairs needed further surgery (*p* = 1).

**Table 3:** Repeat surgery after index repair

	Perigee (*n* = 48)	Native tissue (*N* = 25)	*P* value
Midurethral sling	5	2	1
Posterior repair	4	4	1
Native anterior repair	0	1	1
Abdominal sacrocolpopexy	4	1	0.65
Division vaginal adhesions	2	0	0.54
Incision introitus	1	0	1
Abdominal paravaginal repair	1	0	1
Excision erosion	2	0	0.54
Vaginal mesh repair	0	2	0.11

## Discussion

More women in the anterior vaginal wall mesh group scored their prolapse as “very much better” or “much better” following surgery compared to those in the native tissue arm. The reoperation rate after the index procedure was similar in both groups with 33% in the mesh group and 32% in the native tissue group undergoing further surgery for either de novo incontinence or recurrent prolapse during follow-up. The rate of mesh erosion was 11.6% although the need for surgical intervention was low (4%). This study is unusual in that it only includes women treated for recurrent prolapse and has a long follow-up period.

The long-term data on vaginal mesh repairs remains difficult to evaluate. There are few long-term series published, and they are heterogeneous involving primary and secondary cases, varying compartments and concomitant procedures [10–12], with varying follow-up. Long-term studies assessing a single procedure in a discrete population remain scarce. Heinonen published a long-term series with a similar follow-up period and protocol to the current study. Their study group was a mix of primary and recurrent (46%) prolapses in either the anterior, posterior or both compartments [12]. After a median of 7 years, they described a mesh erosion rate of 23% with 80% of women being satisfied with the procedure. Lo et al. described a subjective cure rate of 88.6% 3 years after surgery but combined their mesh repairs with a sacrospinous fixation [13]. Karmakar (2015) published a series of women with a 2-year median follow-up. They described cure rates (no anatomical recurrence or symptoms) of 91% in those with mesh repair for recurrent cystocele for an extrusion rate of 21% [11].

Mesh erosion rates were 11.6% with only two women in the study requiring surgical management for mesh erosion (4% of all women). This erosion rate was modest and it was expected that it might be higher in view of the length of follow-up and the fact that all the repairs were repeat procedures for a failed primary native tissue repair. The rate of mesh erosion described in this study is similar to that reported in the Cochrane review (12%) [6] and an equivalent rate to those from the Austrian database of 726 vaginal meshes who found mesh exposure of 12% at 1 year [14]. However, the current study has a much longer follow-up and would be expected to have a higher erosion rate.

There are additional risks unique to mesh repairs including mesh exposure and a longer operating time [6, 13]. The Cochrane 2016 found that by combining operation rates for mesh exposure, repeat prolapse surgery and surgery for stress incontinence, there was a higher reoperation rate in mesh repairs compared with native tissue repairs [6]. This was not found in the current study with no statistically significant difference in overall reoperation rate by this calculation. The MHRA concluded in 2014 that for the majority of women, the use of vaginal mesh implants is safe and effective [15].

Interestingly, despite some high-profile reports in the press, the women in this study were mostly unaware of any reports of complications relating to mesh. Only 19% of the women questioned reported being aware of concerns regarding vaginal mesh repair prior to being contacted for this study. Most of the women had been made aware by a family of a friend who had themselves suffered a complication of vaginal mesh repair. Overall, awareness of mesh controversy was low in this population but may be higher in a different patient group.

A high proportion of the women stopped sexual activity after the repair performed in this study. Discontinuation of sexual activity was similar in both the anterior mesh group and the native tissue repair with 46% of the anterior mesh group becoming sexually inactive and 40% of the native tissue repair becoming sexually inactive. Although sexual activity was noted in the study, much more information would have been obtained if a validated sexual function questionnaire had been used. Unfortunately, this was not a focus of research when the initial surgery was performed up to 8.8 years before this study was performed.

There are certain criticisms that could be made of this study. The study size might not appear large when compared to other studies, but many other studies contain primary cases and short follow-up. The post hoc power calculation suggested that the study might be slightly underpowered to show a difference in the PGI-I although this was not born out in the study results. No pre-operative questionnaires were available for direct comparison of symptoms and so there may be some reporting bias especially due to the long follow-up period. The follow-up time was significantly longer in the mesh arm despite attempts to match the cohorts. This might result in lower satisfaction in the mesh arm due to the need for further surgery for recurrence or new complications found later in the follow-up period. The results suggest the reverse with a lower symptomatic recurrence in the mesh arm. It may therefore be possible that the benefits of mesh might be underestimated due to the longer follow-up in this group. This was a single-center single-surgeon retrospective study and this may limit generalisability. However, in the absence of high-quality data from randomised controlled trials, this remains the best evidence currently available. The follow-up rate in the mesh cohort was excellent with 48/50 women seen (96%). The follow-up in the colporrhaphy group was similar to commonly seen in long-term studies with 66% contacted but this might introduce bias. The results presented concentrate on subjective data with the PGI-I but more qualitative data would have been useful.

## Conclusions

The authors of this study stopped using anterior vaginal wall mesh procedures after 2011 as the operation was perceived to be risky and of no definite benefit. On the basis of the findings of this study, this decision should be reviewed. In the hands of the authors, the anterior mesh repair operation has a durable success rate with low morbidity and is probably superior to a native tissue repair. Unfortunately, this specific mesh is no longer marketed. The erosion rate is similar to studies with shorter follow-up. There may, given these findings, be a role for an anterior vaginal mesh repair performed by a trained surgeon in the carefully counselled woman.

## Abbreviations

PGI-I: Patient global impression of improvement
POP: Pelvic organ prolapse
